# Prognostic impact of weight loss during radiation therapy in patients with head and neck cancer: A systematic review

**DOI:** 10.1177/02601060261419743

**Published:** 2026-02-13

**Authors:** Emelie von Wascinski, Julijana Vracaric, Klaus-Henning Kahl, Stefan Schiele, Anna Rubeck, Lukas Käsmann, Johannes Doescher, Johannes Zenk, Georg Stüben, Maria Neu

**Affiliations:** 1 Department of Radiotherapy and Radiation Oncology, Faculty of Medicine, 26522 University of Augsburg, Augsburg, Germany; 2 Institute of Philosophy, Faculty of Philosophy and Social Sciences, 39694 University of Augsburg, Augsburg, Germany; 3 Comprehensive Cancer Center Augsburg (CCCA), Faculty of Medicine, 39694 University of Augsburg, Augsburg, Germany; 4 Comprehensive Cancer Center Alliance WERA (CCC WERA), Augsburg, Germany; 5 Bavarian Cancer Research Center (BZKF), Augsburg, Germany; 6 Institute of Mathematics, 26522 University of Augsburg, Augsburg, Germany; 7 Department of Radiation Oncology, 9183 University Hospital, LMU Munich, Munich, Germany; 8 German Cancer Consortium (DKTK), Partner Site Munich, Munich, Germany; 9 Bavarian Cancer Research Center (BZKF), Munich, Germany; 10 LA Regio Kliniken, Klinik Landshut - Mitte, Department of Radiation Oncology, Landshut, Germany; 11 Department of Otorhinolaryngology and Head & Neck Surgery, University Hospital Augsburg, Augsburg, Germany

**Keywords:** Malnutrition, nutritional management, treatment outcome, body mass index, radiation-induced toxicity, supportive care

## Abstract

**Background:**

Malnutrition and weight loss (WL) are frequent in patients with head and neck cancer (HNC) during radiotherapy (RT), affecting treatment tolerance and outcomes. Nutritional interventions aim to minimize WL and support therapy completion, yet the prognostic value of WL during RT remains unclear.

**Aims/Objectives:**

To systematically evaluate the prognostic impact of WL before, during and after RT in patients with HNC.

**Methods/Methodology:**

This systematic review included studies from 2012 involving adult patients treated with definitive or postoperative RT for HNC, studies were eligible if WL/body mass index (BMI) change was analysed versus survival outcomes (overall survival (OS), disease-specific survival (DSS)/cancer-specific survival, disease-free survival (DFS)). A structured PubMed and Cochrane search was conducted and results were synthesized narratively.

**Results/Findings:**

Eight studies met the inclusion criteria. Pretreatment WL > 10% consistently predicted inferior OS and disease-specific survival (DSS). WL during RT varied widely between studies: most reported no association with OS, whereas single studies reported worse DSS with critical WL, worse OS with ΔBMI >1 kg/m^2^, or an apparent survival advantage with greater WL. Posttreatment WL ≥ 10% was associated with reduced DFS. Comparability was limited by heterogeneous WL definitions, timing and treatment techniques.

**Conclusion:**

Pretreatment WL is a strong negative prognostic factor in HNC, whereas evidence for WL during or after RT remains inconsistent. Standardized WL assessment and structured nutritional support should be integrated into routine RT care. Future prospective studies using harmonized definitions are needed to clarify prognostic relevance and guide evidence-based nutrition management.

## Introduction

Head and neck cancer (HNC) rank as the seventh most common malignancy worldwide and include a heterogeneous group of tumours originating in the paranasal sinuses, nasopharynx, oral cavity, pharynx (naso-, oro- and hypopharynx) and larynx (Alterio et al., 2019; Mody et al., 2021). Radiotherapy (RT), with or without chemotherapy (CTX) or surgery, plays a central role in curative treatment, particularly in advanced stages (Alterio et al., 2019).

Despite therapeutic advances, patients undergoing RT or chemoradiotherapy often experience severe side effects such as mucositis, dysphagia and dermatitis, which frequently lead to weight loss (WL) and malnutrition (Ghadjar et al., 2015; Han et al., 2021; Löser et al., 2024). While pretreatment nutritional status is recognized as a prognostic factor, the impact of WL occurring during RT on overall survival (OS) and disease-free survival (DFS) remains unclear (Finger et al., 2024). WL in HNC is not only driven by reduced intake due to mucositis and dysphagia but can also reflect systemic inflammation and tumour-related catabolism. These mechanisms may reduce physiologic reserve, impair treatment tolerance and affect survival. This systematic review aims to synthesize the available evidence on the prognostic value of WL alongside RT in patients with HNC.

## Patients and methods

This systematic review was conducted in accordance with the Preferred Reporting Items for Systematic Reviews and Meta-Analyses (PRISMA) guidelines; all eligibility criteria, data extraction steps and outcomes were predefined to minimize selection and reporting bias. The review protocol was not registered (e.g. PROSPERO). A structured search of PubMed/MEDLINE and the Cochrane Library was conducted in November 2024, using Medical Subject Headings (MeSH) and Title/Abstract terms related to *head and neck cancer*, *radiotherapy*, *nutrition*, *weight loss* or *body mass index (BMI)*, and *survival/prognosis*. The full electronic search syntax is provided in Appendix. Embase and Scopus were not searched; this is a limitation of the review.

Studies published from 2012 onward were included to ensure comparability with contemporary standards of HNC care. Around this time, intensity-modulated radiotherapy (IMRT) and volumetric modulated arc therapy (VMAT) became routine, replacing older 3D-conformal techniques and substantially changing dose distribution, toxicity profiles and nutritional impact. Because IMRT/VMAT allow better sparing of swallowing-related structures and salivary glands, they may reduce dysphagia- and xerostomia-driven reductions in oral intake compared with older 3D techniques. Therefore, restricting inclusion to studies from 2012 onward improves clinical comparability of treatment toxicity and its nutritional consequences. In parallel, concurrent cisplatin-based chemoradiotherapy was the established standard of care, and structured nutritional screening and support protocols were increasingly implemented in clinical practice. Eligibility criteria included: (i) histologically confirmed HNC; (ii) treatment with RT or chemoradiotherapy; (iii) human studies; (iv) quantitative assessment of WL, defined either in kilograms (kg) or via BMI change; (v) reporting of survival outcomes. Exclusion criteria comprised case reports, reviews, letters, editorials and duplicates.

### Definitions and terminology

To ensure consistency across studies, the following terminology was applied throughout the review:

- *WL:* any documented reduction in body weight or BMI relative to baseline, expressed either as percentage of initial body weight or as ΔBMI in kg/m^2^.
- *Critical WL:* a loss >5% within 8 weeks or >7.5% within 12 weeks, according to the international consensus by White et al. (2012)
- *Pretreatment WL:* WL occurring within 6 months before the start of RT.
- *WL during RT:* WL documented between the first and last day of RT or chemoradiotherapy.
- *Posttreatment WL:* WL measured after completion of RT, up to 6 months post-therapy.

**Figure 1.**
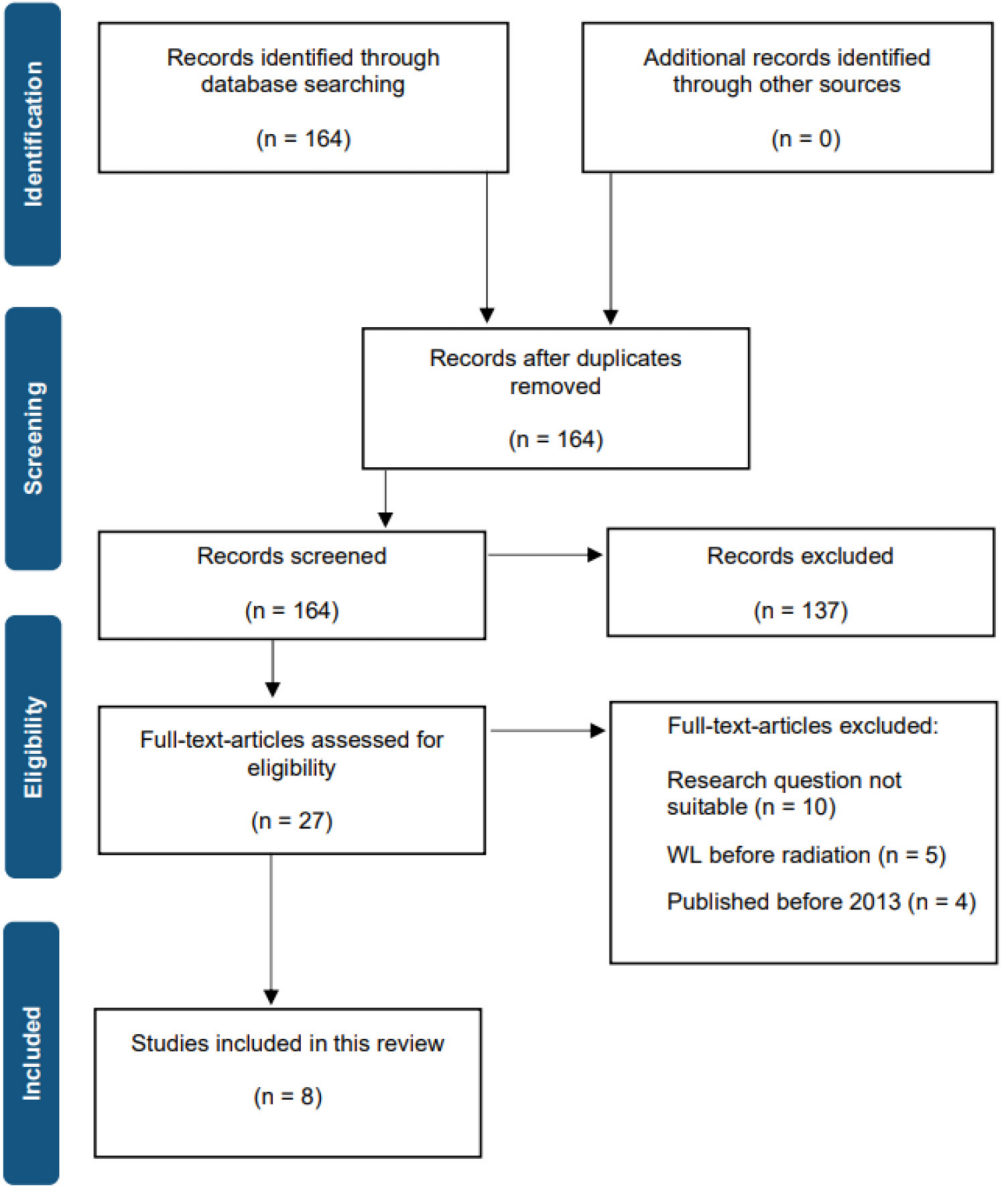
PRISMA flow diagram of literature search and study selection; WL: weight loss.

Where individual studies used alternative definitions, data were standardized to the extent possible for descriptive comparison. Data extraction focused on study characteristics, treatment details, WL metrics and survival outcomes. We considered limited subgroup pooling (e.g., pretreatment WL >10% and ΔBMI >1 kg/m^2^), but meta-analysis remained inappropriate due to substantial heterogeneity and incomplete reporting of effect estimates. Definitions of intratreatment WL ranged from ≥5% to ≥10%, and assessment windows differed (end of RT, 2 months post-RT, or up to 5 months post-RT), alongside variation in endpoints (OS, DSS/cancer-specific survival (CSS), DFS, progression-free survival (PFS), distant metastasis-free survival (DMFS)/locoregional recurrence-free survival (LRRFS)). Because effect estimates and time points were not sufficiently comparable, statistical heterogeneity metrics (e.g., I^2^) were not calculated. A PRISMA flow diagram is detailed in Figure 1.

The methodological quality of all included studies was assessed independently by two reviewers using the Newcastle–Ottawa Scale (NOS) for cohort studies. When details were unclear (e.g., handling of missing data, exact follow-up time), conservative assumptions were applied to avoid overestimation of study quality. Inter-rater agreement between the two reviewers was κ = 0.84, indicating strong concordance, disagreements were resolved by consensus. A summary of the NOS ratings is provided in Supplementary Table S1.

Quantitative pooling of data was deemed inappropriate because of marked heterogeneity among studies regarding tumour subsites, treatment modalities, weight-loss definitions and survival endpoints. A narrative synthesis was therefore conducted. However, where studies shared comparable definitions (e.g., pretreatment WL > 10%), results were examined descriptively to identify consistent patterns. Because no quantitative pooling was performed, studies were not statistically weighted by sample size. In the narrative synthesis, we placed greater emphasis on larger cohorts and higher-quality studies when deriving overall conclusions.

## Results

Following a comprehensive review of the extant literature, 164 articles were identified as potentially relevant. However, subsequent to the application of the inclusion criteria, eight articles were determined to meet all the specified criteria and thus were included in the final analysis. The eight articles incorporated both retrospective and prospective designs. The included studies analysed various tumour subsites (e.g., nasopharynx, oropharynx, oral cavity), RT techniques (e.g., IMRT and VMAT), WL definitions and endpoints. This provides a broad perspective on the prognostic relevance of WL in modern HNC care. Table 1 provides a summary of key study characteristics and outcomes.

**Table 1.** Overview of included studies on the prognostic impact of weight loss (WL) and BMI in head and neck cancer.

Author (year)	Tumour entity/RT setting	Outcomes	WL definition (timing/threshold)	*N* (population details)	Key findings
Langius et al. (2013)	Mixed HNC (RT ± surgery ± CTX)	5-year OS; DSS	Pre-WL > 10% (≤6 mo); WL during RT > 5% ≤ 8 wk or >7.5% ≤ 12 wk	1340	Pre-WL > 10% → worse OS (HR 1.7; *P* = 0.002) and DSS (HR 2.1; *P* = 0.007). WL during RT > 5% → worse DSS (HR 1.7; *P* = 0.004) but not OS
Ghadjar et al. (2015)	Locally advanced HNC (hyperfractionated RT ± cisplatin)	5-year OS, DMFS, LRRFS, CSS	Pre-WL ≥ 10% (≤6 mo); WL during RT ≥ 10%	224	Pre-WL ≥ 10% → worse OS (HR 3.2; *P* < 0.001), LRRFS (HR 2.5; *P* < 0.001), DMFS (HR 3.1; *P* < 0.001), CSS (HR 2.8; *P* = 0.002). WL during RT→ no impact.
Han et al. (2021)	Mixed HNC (definitive CRT 71%, post-op RT 29%)	Median OS; 5-year OS; CSS	WL during RT ≥ 5.8% (dichotomized at median)	843	High WL (≥5.8%) → better OS (HR 0.75; *P* = 0.01) and CSS (HR 0.71; *P* = 0.01). Interpretation likely affected by confounders.
Moon et al. (2016)	HNSCC (definitive CRT + high-dose cisplatin)	OS; CSS; PFS	Pre-BMI < 18.5 kg/m^2^; WL during RT (ΔBMI pre-vs 2 month post-RT)	153	Low Pre-BMI < 18.5 → poor OS (HR 15.2; *P* < 0.001) and CSS (HR 20.1; *P* < 0.001). WL during RT → no association.
Lin et al. (2015)	nasopharyngeal carcinoma (IMRT ± CTX)	3-year OS; DSS	WL during RT ≥ 5%; Pre-BMI < 23 kg/m^2^	238	WL during RT ≥ 5% → no impact on OS (*P* = 0.087) or DSS (*P* = 0.232). Pre-BMI < 23 → no effect.
Lin et al. (2022)	nasopharyngeal carcinoma (VMAT ± CTX)	5-year OS; DFS; D-FFR; ΔBMI	During RT-ΔBMI > 1 kg/m^2^; Pre-BMI categories	498	ΔBMI > 1 kg/m^2^ → lower 5-yr OS (73.0% vs 88.0%; *P* < 0.001), DFS (65.1% vs 80.4%; *P* < 0.001), D-FFR (78.5% vs 90.9%; *P* < 0.001). Independent predictor of poor outcome.
Cho et al. (2013)	Oral / oropharyngeal SCC (mixed modalities)	DFS	Post-WL ≥ 10% (≤6 month post-RT)	226	Post-WL ≥ 10% → reduced DFS (*P* = 0.036); independent predictor of poor DFS. OS not reported.
Ottosson et al. (2014)	oropharyngeal carcinoma (ARTSCAN trial)	5-year OS	WL during RT ≥ 10% (start RT → 5 mo); Pre-BMI > 25 kg/m^2^	357	WL during RT ≥ 10% → no impact on 5-yr OS (*P* = 0.708). Pre-BMI > 25 kg/m^2^→ better OS (*P* < 0.05). WL correlated with irradiated volume.

WL: weight loss; Pre-WL: pretreatment weight loss within 6 months before RT; WL during RT: weight loss assessed during RT or in the early post-RT period, as defined in the individual studies; Post-WL: weight loss within 6 months after RT; ΔBMI: change in body mass index (kg/m^2^) from baseline to the study-specific assessment time point (end of RT or early post-RT, as reported); BMI: body mass index; OS: overall survival; HR: hazard ratio; DSS: disease-specific survival; DFS: disease-free survival; CSS: cancer-specific survival; PFS: progression free survival; LRRFS: locoregional recurrence-free survival; DMFS: distant metastasis-free survival; D-FFR: distant failure-free rate; RT: radiotherapy; CRT: chemoradiotherapy; CTX: chemotherapy; HNC: head and neck cancer; HNSCC: head and neck squamous cell carcinoma; VMAT: volumetric modulated arc therapy.

**Figure 2.**
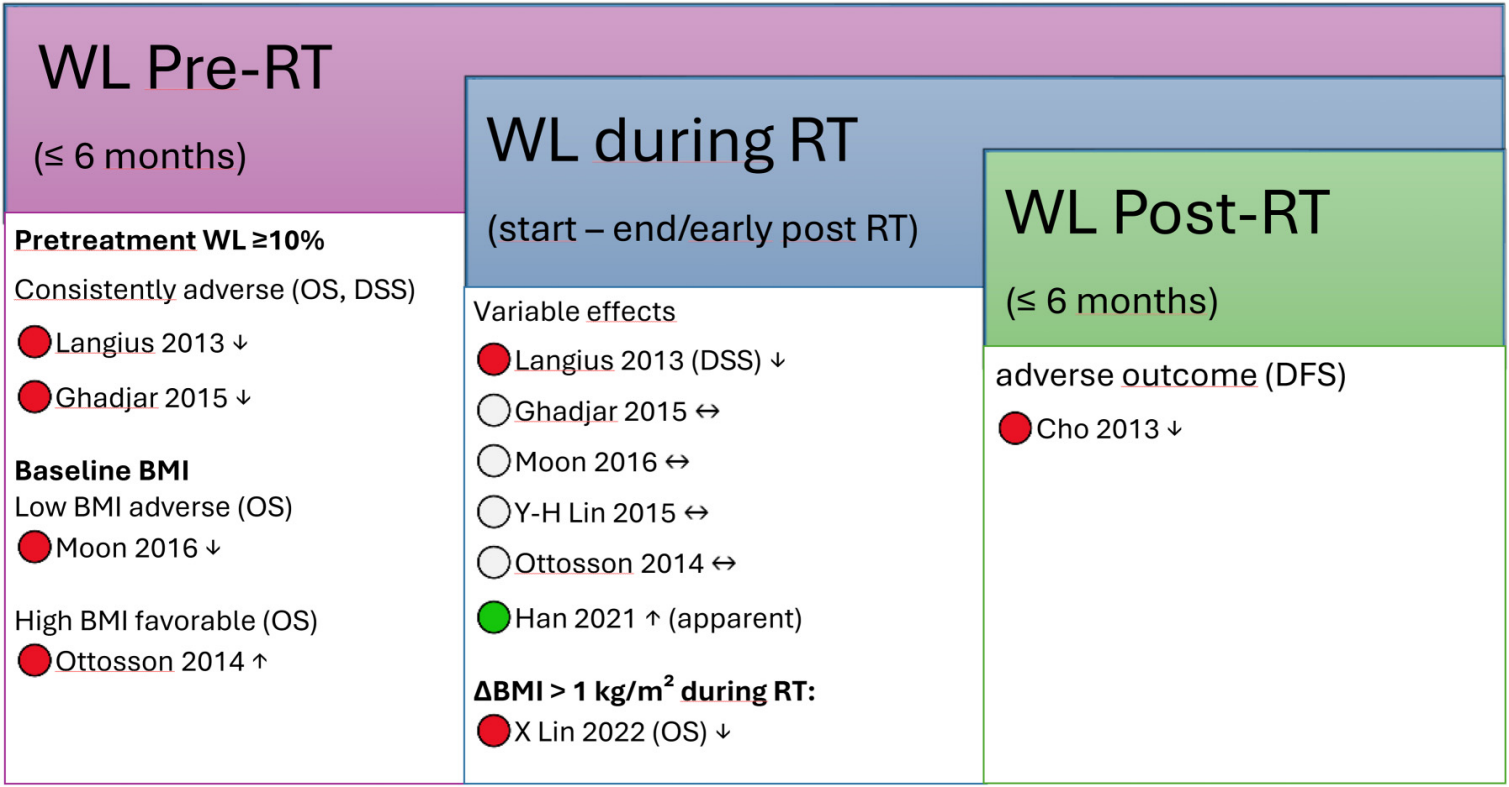
Timeline of weight-loss assessment and corresponding prognostic impact across eight included studies. In the two studies assessing pretreatment WL ≥10% within 6 months before RT, this was consistently associated with worse survival outcomes (OS/DSS) (↓). Baseline BMI showed prognostic relevance, with low BMI associated with adverse OS and higher BMI associated with better OS. WL during RT/early post-RT showed variable effects across studies: most reported no association (↔), two reported adverse associations (one for DSS and one for OS/DFS/D-FFR using ΔBMI) (↓), and one reported an apparent favourable association (↑; likely confounding). Posttreatment WL within 6 months after RT was associated with reduced DFS (↓). Arrows indicate direction of association (↓ adverse, ↔ no association, ↑ apparent favourable).

An overview of the timing and direction of associations is illustrated in Figure 2.

*Langius* et al. conducted a prospective analysis including 1340 patients with HNC treated with curative intent using RT, either alone or in combination with surgery ± CTX. The study investigated the prognostic significance of WL before and during RT on 5-year OS and disease-specific survival (DSS). Critical WL during RT was defined, in accordance with the international consensus statement by White et al. (2012), as a body weight reduction of >5% from the start of RT until week 8, or >7.5% until week 12. This condition was observed in 57% of patients. The mean WL during RT was 4.1 kg (±4.7), corresponding to 5.4% (±6.1%) of baseline body weight. Unadjusted 5-year OS and DSS were significantly lower in patients with critical WL during RT compared to those without (OS: 62% vs. 70%, *P* = 0.01; DSS: 82% vs. 89%, *P* = 0.001). In multivariable analysis, critical WL remained significantly associated with impaired DSS (hazard ratio [HR] 1.7, 95% CI [1.2–2.4], *P* = 0.004), but not with OS (HR 1.1, 95% CI [0.9–1.4], *P* = 0.295). Additionally, pre-treatment WL showed a dose-dependent prognostic effect: while 70% of patients had no WL before RT, 16% had WL ≤5%, 9% had 5–10% and 5% had >10% WL. Corresponding 5-year OS rates declined with increasing WL (71%, 59%, 47% and 42%, respectively; *P* < 0.001), and the DSS rates followed a similar trend (86%, 86%, 81% and 71%; *P* < 0.001). In adjusted models, pre-treatment WL >10% was independently associated with worse OS (HR 1.7, 95% CI [1.2–2.5], *P* = 0.002) and DSS (HR 2.1, 95% CI [1.2–3.5], *P* = 0.007) (Langius et al., 2013).

*Ghadjar* et al. analysed 224 patients with locally advanced head and neck squamous cell carcinoma (HNSCC) enrolled in the randomized phase III SAKK 10/94 trial. Patients were treated with hyperfractionated RT, either alone or in combination with cisplatin-based CTX. The study evaluated the prognostic value of WL both before and during treatment. WL was measured retrospectively for the 6 months prior to therapy and prospectively during treatment. A threshold of ≥10% WL prior to treatment initiation was identified as clinically relevant. In multivariable analyses, pretreatment WL ≥10% was significantly associated with worse outcomes across all survival endpoints, including OS (HR 3.2, 95% CI [2.0–5.3], *P* < 0.001), LRRFS (HR 2.5, 95% CI [1.4–4.5], *P* < 0.001), DMFS (HR 3.1, 95% CI [1.6–6.1], *P* < 0.001) and CSS (HR 2.8, 95% CI [1.5–5.4], *P* = 0.002). In contrast, WL during treatment – although common (occurring in 51% of patients) – was not significantly associated with any of the analysed survival endpoints in either univariable or multivariable analysis. The authors emphasize the need for early nutritional intervention and vigilant monitoring prior to the initiation of treatment (Ghadjar et al., 2015).

*Han* et al. conducted a retrospective single-centre analysis of 843 patients with HNC who received RT with curative intent, either definitively (71%) or postoperatively (29%), between 2003 and 2017. WL during treatment was assessed as a continuous variable, with the cohort dichotomized at the median WL of 5.8%. Patients with high WL (≥5.8%) showed significantly improved median OS (39.2 vs. 36.7 months; *P* = 0.047) and 5-year OS (54.9% vs. 48.8%; *P* = 0.047) compared to patients with lower WL. Similarly, 5-year CSS was higher in the high WL group (64.0% vs. 58.2%; *P* = 0.036). In multivariate Cox regression, high WL was independently associated with improved OS (HR 0.75, 95% CI [0.61–0.93], *P* = 0.01) and CSS (HR 0.71, 95% CI [0.55–0.93], *P* = 0.01) (Han et al., 2021).

*Moon* et al. conducted a prospective study involving 153 patients with HNSCC undergoing definitive chemoradiotherapy. Body weight and BMI were recorded within 2 weeks before and 2 months after treatment. Although a significant reduction in weight and BMI was observed during therapy, neither WL nor post-treatment BMI change showed a significant association with PFS, CSS, or OS. In contrast, low pretreatment BMI (<18.5 kg/m^2^) was independently associated with worse survival outcomes, including CSS (HR 20.12, 95% CI [4.82–83.96], *P* < 0.001) and OS (HR 15.23, 95% CI [4.06–57.11], *P* < 0.001). Additional predictors of adverse outcome included pretreatment hypoalbuminemia and elevated neutrophil-to-lymphocyte ratio (NLR), while changes in body weight alone were not found to be prognostically relevant (Moon et al., 2016).

*Yu-Hsuan Lin* et al. performed a retrospective study on 238 patients with nasopharyngeal carcinoma (NPC) treated with IMRT. The prognostic value of pretreatment BMI and WL during therapy was evaluated. Patients were stratified by BMI (<23 vs. ≥23 kg/m^2^) and WL (≥5% vs. <5%), with 63% of the cohort experiencing WL ≥5% during treatment. However, neither parameter was significantly associated with OS or DSS in univariate or multivariate analysis. Specifically, WL ≥5% showed no negative impact on 3-year OS (*P* = 0.087) or DSS (*P* = 0.232), and BMI stratification showed no prognostic value (OS: *P* = 0.672; DSS: *P* = 0.341). Subgroup analyses also revealed no interaction between baseline BMI and the magnitude of WL. Sensitivity analyses using different cutoff values for BMI and WL confirmed these findings (Lin et al., 2015).

*Xiang Lin* et al. conducted a retrospective single-centre study of 498 patients with NPC treated with VMAT. The prognostic relevance of pretreatment BMI and treatment-related BMI change (ΔBMI) was assessed. Pretreatment BMI showed no statistically significant association with OS, DFS, distant failure-free survival (D-FFR), or locoregional control. In contrast, a decrease in BMI >1 kg/m^2^ during treatment was significantly associated with inferior oncologic outcomes. Patients with ΔBMI >1 kg/m^2^ had significantly lower 5-year OS (73.0% vs. 88.0%, *P* < 0.001), DFS (65.1% vs. 80.4%, *P* < 0.001) and D-FFR (78.5% vs. 90.9%, *P* < 0.001) compared to those with ΔBMI ≤1 kg/m^2^. Multivariate analysis confirmed ΔBMI >1 kg/m^2^ as an independent predictor of poor outcome, whereas pretreatment BMI alone showed no prognostic significance. The authors concluded that ΔBMI is a simple and clinically accessible marker to identify patients at increased risk and emphasized the need for timely nutritional intervention in this population (Lin et al., 2022).

*Cho* et al. conducted a retrospective study analysing 226 patients with previously untreated, operable squamous cell carcinoma (SCC) of the oral cavity (*n* = 123) or oropharynx (*n* = 103), treated at a single centre between 2005 and 2010. Patients underwent heterogeneous treatment modalities including surgery, RT, CTX, or combinations thereof. Body weight was measured at several time points, and the first day of treatment was defined as the baseline. Posttreatment WL ≥10% was observed in 24.2% of patients and was attributed to both tumour-related and treatment-induced factors such as RT, disease recurrence and the presence of ≥3 metastatic lymph nodes. Multivariate logistic regression identified RT, posttreatment recurrence and multiple nodal metastases as independent predictors of WL ≥10%. Additionally, significant WL was associated with worse DFS (*P* = 0.036). In multivariate analysis, WL ≥10% remained an independent predictor of poor DFS. OS was not reported (Cho et al., 2013).

*Ottosson* et al. conducted a retrospective cohort study using data from 357 patients with oropharyngeal carcinoma enrolled in the ARTSCAN trial. The study investigated the prognostic significance of WL and BMI in relation to 5-year OS, as well as the association between WL and irradiated volume. WL was defined as the percentage weight change from the start of RT to 5 months post-treatment and dichotomized at a 10% threshold. WL ≥10% occurred in 68% of evaluable patients but was not significantly associated with 5-year OS (77.0% vs. 74.1%; *P* = 0.708). In contrast, baseline BMI >25 kg/m^2^ was significantly associated with improved survival outcomes. Patients classified as overweight or obese had a 5-year OS of 83.2%, compared to 58.8% and 56.7% in the underweight and normal-weight groups, respectively. In multivariate Cox regression, BMI remained an independent prognostic factor (HR 3.78 for underweight, 95% CI [1.46–9.75]; HR 2.57 for normal weight, 95% CI [1.43–4.62]). Moreover, irradiated volume (TV64.6 Gy and TV43.7 Gy) was significantly correlated with increased WL, highlighting the importance of radiation dose distribution in nutritional outcomes (Ottosson et al., 2014).

Across the two largest high-quality cohorts, pretreatment WL ≥10% showed a consistent association with worse survival, with reported hazard ratios ranging from HR 1.7 to HR 3.2 for OS and from HR 2.1 to HR 2.8 for DSS/CSS. A detailed evidence map is provided in Supplementary Table S2, an overview matrix summarizing prognostic associations by WL timing and outcome is provided in Supplementary Table S3.

## Discussion

This systematic review summarizes eight studies assessing the prognostic significance of WL before, during and after RT in HNC. Across studies, pretreatment WL >10% consistently predicted poorer overall and disease-specific survival, whereas the impact of WL during or after RT was less uniform. Methodological heterogeneity and inconsistent WL definitions likely explain the divergent results.

### Pretreatment WL

Pretreatment WL (Pre-WL) consistently proved to be a strong negative prognostic factor. In two large and methodologically robust studies, Langius et al. and Ghadjar et al. demonstrated that Pre-WL >10% within 6 months before therapy initiation independently predicted reduced overall and disease-specific survival. These findings indicate that early nutritional depletion mirrors tumour burden and reduced physiologic reserve. Similarly, Moon et al. and Ottosson et al. reported that a low baseline BMI (<18.5 kg/m^2^) or underweight status was associated with significantly poorer survival, reinforcing the relevance of early nutritional screening and optimization before treatment.

### WL during RT

The prognostic value of WL during RT was inconsistent across studies. Langius et al. found that During-WL >5% within 8 weeks was associated with reduced disease-specific survival but not OS. Xiang Lin et al. observed that a BMI decrease >1 kg/m^2^ during VMAT was an independent predictor of inferior OS, DFS and D-FFR. In contrast, Ottosson et al., Yu-Hsuan Lin et al. and Moon et al. reported no prognostic impact. Han et al. described an apparent association between greater WL during RT (≥5.8%) and improved survival; however, baseline BMI and pretreatment WL were not available, and WL was dichotomized at the cohort median. Residual confounding (e.g., baseline body composition, treatment intensity, smoking, comorbidity) and selection effects may explain this finding, and the remaining studies do not support a beneficial effect of WL during RT. Overall, these heterogeneous findings suggest that WL during RT may not directly influence prognosis but rather reflect treatment-related inflammation, hydration changes, or temporary nutritional compromise.

### Posttreatment WL

Only Cho et al. analysed posttreatment WL (Post-WL), identifying Post-WL ≥10% within 6 months after therapy as an independent predictor of poor DFS. This indicates that persistent or progressive weight decline after RT may reflect ongoing catabolism, disease recurrence, or late toxicity, warranting close nutritional and clinical follow-up in survivorship care.

### Potential confounders and mechanisms

Several factors may confound the link between WL and survival, including smoking, alcohol use, comorbidities, systemic inflammation and variable treatment tolerance. Inflammatory activity (e.g., elevated CRP or NLR) can drive catabolism and WL independent of caloric intake. Low baseline albumin and haemoglobin are associated with worse outcomes, but they largely reflect inflammation and disease burden rather than nutrient intake; thus, laboratory markers should be interpreted together with clinical and anthropometric data.

Current diagnostic frameworks, including the GLIM criteria, recommend combining phenotypic indicators (e.g., WL, low BMI, reduced muscle mass) with etiologic factors (reduced intake, inflammation) for a valid diagnosis of malnutrition (Cederholm et al., 2019). Intentional, lifestyle-related WL should also be differentiated from cancer- or therapy-induced cachexia, as their underlying mechanisms and prognostic implications differ.

Beyond body weight, CT-based assessment of skeletal muscle has gained increasing attention in HNC. Recent work has proposed approaches to quantify the severity of sarcopenia in this population, highlighting that clinically relevant loss of muscle may not be captured by WL alone (Kubrak et al., 2024). In addition, a systematic review and meta-analysis showed that sarcopenia is associated with higher short-term treatment-related toxicity in patients receiving curative-intent therapy for HNC (Mascarella et al., 2024). A further systematic review in patients treated with definitive (chemo-)radiotherapy reported mixed prognostic findings, likely reflecting variability in measurement methods and sarcopenia definitions (Vickers et al., 2025). Together, these data suggest that WL during RT should be interpreted in the context of body composition and inflammation-related catabolism and translated into structured nutritional monitoring and supportive care pathways.

### Clinical implications and guideline context

These findings align with ESPEN guidance for patients receiving RT, especially in HNC. ESPEN recommends regular screening from diagnosis onward and, in case of abnormal screening, objective assessment of intake, symptoms, muscle mass, performance and inflammation (Muscaritoli et al., 2021). Early nutrition care should begin with individualized counselling and oral nutritional supplements (ONS); if oral intake remains inadequate, enteral nutrition (EN) is indicated, while parenteral nutrition (PN) should be reserved for patients in whom EN is not feasible. In HNC, prophylactic or early reactive EN via nasogastric tube or PEG may help maintain nutritional status and treatment continuity.

### Limitations

This review included only eight studies, most of them retrospective and heterogeneous regarding tumour sites, treatment techniques, WL definitions and outcome measures. Key prognostic variables such as HPV status, comorbidity burden and systemic inflammation were inconsistently reported. These differences precluded meta-analysis and limited comparability. Nonetheless, consistent signals for the prognostic value of Pre-WL strengthen confidence in the findings. Although nutritional management practices have evolved over the last decade, most studies did not report the use or timing of dietetic support, oral nutritional supplements (ONS), or enteral feeding, which limits assessment of the actual nutrition dose delivered. Publication bias cannot be excluded. Because only eight heterogeneous observational studies were included, formal assessment (e.g., funnel plots) was not meaningful. Smaller studies with null findings may be underrepresented. We did not apply GRADE because included studies were observational and highly heterogeneous; NOS was used as the primary quality tool.

### Future directions

*Standardization of definitions:* Future studies should adopt harmonized criteria for defining and reporting WL, such as ≥5% within 1 month or ≥10% within 6 months, or express changes as ΔBMI > 1 kg/m^2^. Reporting the exact assessment time points (pretreatment, during-treatment, post-treatment) is essential to enable comparison across studies.

*Adjustment for key confounders and integration of biomarkers:* Prospective data collection should include baseline BMI, comorbidities, systemic inflammation markers (CRP, albumin, prealbumin, lymphocyte count) and treatment intensity to allow robust multivariate analysis and distinction between cachexia and general malnutrition.

*Functional and patient-reported outcomes:* Future studies should complement WL/BMI with body composition and function, including CT-derived muscle measures (e.g., skeletal muscle index) and, where feasible, longitudinal muscle change during RT. Standardized approaches to quantify sarcopenia severity and agreed definitions would improve comparability across cohorts and help clarify whether prognosis is driven by WL per se or by loss of muscle mass (Kubrak et al., 2024; Vickers et al., 2025). Given the association between sarcopenia and short-term treatment-related toxicity, integrating baseline sarcopenia into supportive care pathways may also help identify patients at risk of poor tolerance and treatment interruptions (Mascarella et al., 2024).

*Prehabilitation and supportive interventions:* Research should assess multimodal prehabilitation programs combining nutritional counselling, exercise and psychosocial support before and during RT. Early evidence in other tumour entities suggests that such approaches can improve functional capacity and treatment tolerance; their value in HNC remains to be established.

*Implementation research:* Beyond prognostic associations, pragmatic trials should evaluate how structured nutritional screening and early dietetic referral influence real-world outcomes such as unplanned hospitalizations, treatment completion and quality of life.

Collectively, these directions will help clarify the biological mechanisms underlying WL, standardize data reporting and guide the development of evidence-based nutritional strategies in head and neck RT.

## Conclusion

This systematic review highlights heterogeneous evidence on WL as a prognostic marker in HNC. While pretreatment WL is consistently associated with worse outcomes across multiple studies, the prognostic significance of WL during and after RT remains variable. The timing and context of WL appear to influence its clinical relevance, with early nutritional depletion posing greater prognostic concern than treatment-related changes alone. Future research should focus on harmonizing definitions, improving nutritional risk assessment, and evaluating structured interventions to support patients with or at risk of critical WL. Integrated nutritional care should be considered an essential component of multidisciplinary oncologic management. Standardized screening at defined time points could support early dietetic referral and help identify patients who may benefit from intensified supportive care during RT.

## Supplemental Material

sj-docx-1-nah-10.1177_02601060261419743 - Supplemental material for Prognostic impact of weight loss during radiation therapy in patients with head and neck cancer: A systematic review Supplemental material, sj-docx-1-nah-10.1177_02601060261419743 for Prognostic impact of weight loss during radiation therapy in patients with head and neck cancer: A systematic review by Emelie von Wascinski, Julijana Vracaric, Klaus-Henning Kahl, Stefan Schiele, Anna Rubeck, Lukas Käsmann, Johannes Doescher, Johannes Zenk, Georg Stüben and Maria Neu in Nutrition and Health

sj-docx-2-nah-10.1177_02601060261419743 - Supplemental material for Prognostic impact of weight loss during radiation therapy in patients with head and neck cancer: A systematic review Supplemental material, sj-docx-2-nah-10.1177_02601060261419743 for Prognostic impact of weight loss during radiation therapy in patients with head and neck cancer: A systematic review by Emelie von Wascinski, Julijana Vracaric, Klaus-Henning Kahl, Stefan Schiele, Anna Rubeck, Lukas Käsmann, Johannes Doescher, Johannes Zenk, Georg Stüben and Maria Neu in Nutrition and Health

## Appendix. Electronic search strategy

*Database searched:* PubMed/MEDLINE (via NCBI) and Cochrane Library

*Date of last search:* November 30, 2024

*Limits:* Human studies, English language, publication year ≥ 2012

*Search fields:* Title/Abstract and MeSH Terms

*Search string:*

((“head and neck neoplasms"[MeSH Terms]) OR (“head and neck cancer"[Title/Abstract]) OR (“HNSCC"[Title/Abstract]))

AND

((“radiotherapy"[MeSH Terms]) OR (“chemoradiotherapy"[Title/Abstract]) OR (“radiation therapy"[Title/Abstract]))

AND

((“weight loss"[MeSH Terms]) OR (“body mass index"[MeSH Terms]) OR (“BMI change"[Title/Abstract]) OR (“malnutrition"[Title/Abstract]) OR (“nutritional status"[Title/Abstract]))

AND

((“survival"[MeSH Terms]) OR (“prognosis"[MeSH Terms]) OR (“overall survival"[Title/Abstract]) OR (“disease-free survival"[Title/Abstract]))

*Additional steps:*

- Manual screening of bibliographies from key papers and reviews.
- Cross-checking in Cochrane Library using equivalent MeSH terms.
- No additional filters (sex, age, tumour site) were applied to maximize sensitivity.
