# Supplementary material for: Prognostic impact of weight loss during radiation therapy in patients with head and neck cancer: A systematic review
Source: Nutr Health. 2026 Feb 13;32(4):1169–78. doi: 10.1177/02601060261419743 (PMC13338475; doi:10.1177/02601060261419743)
Supplement: sj-docx-1-nah-10.1177_02601060261419743 - Supplemental material for Prognostic impact of weight loss during radiation therapy in patients with head and neck cancer: A systematic review [file sj-docx-1-nah-10.1177_02601060261419743.docx]

## Supplementary

***Supplementary*** ***Table S1:*** *Quality Assessment (Newcastle–Ottawa Scale); NOS = Newcastle–Ottawa Scale; High = 7–9 points; Moderate = 5–6 points; Low = <5 points.*

| **Author, year** | **Study Design** | **Selection (0–4)** | **Comparability (0–2)** | **Outcome (0–3)** | **Total Score (0–9)** | **Quality** |
| --- | --- | --- | --- | --- | --- | --- |
| **Langius et al. (2013)** | Prospective cohort | 4 | 2 | 3 | **9** | High |
| **Ghadjar et al. (2015)** | Prospective (secondary analysis of RCT) | 4 | 2 | 3 | **9** | High |
| **Han et al. (2021)** | Retrospective single-center | 3 | 1 | 2 | **6** | Moderate |
| **Moon et al. (2016)** | Prospective observational | 3 | 2 | 2 | **7** | High |
| **Yu-Hsuan Lin et al. (2015)** | Retrospective single-center | 3 | 1 | 2 | **6** | Moderate |
| **Xiang Lin et al. (2022)** | Retrospective single-center | 3 | 2 | 3 | **8** | High |
| **Cho et al. (2012)** | Retrospective single-center | 3 | 1 | 2 | **6** | Moderate |
| **Ottosson et al. (2014)** | Retrospective cohort (ARTSCAN trial) | 4 | 2 | 3 | **9** | High |

***Supplementary*** ***Table S2:*** Evidence map summarizing the timing of weight-loss (WL) assessment, definitions, and prognostic impact across the included studies

| **Author, year** | **WL Timing** | **WL Definition / Threshold** | **Survival Endpoints** | **Direction of Association** | **Statistical Significance** | **Key Comments** |
| --- | --- | --- | --- | --- | --- | --- |
| **Langius et al. (2013)** | **Pre-RT** | WL > 10 % within 6 months | OS, DSS | ↓ (both) | p < 0.01 | Strong negative prognostic factor |
| **Langius et al. (2013)** | **During-RT** | WL > 5 % (≤ 8 wk) or > 7.5 % (≤ 12 wk) | DSS, OS | ↓ DSS; ↔ OS | p < 0.01 (DSS) | Critical WL associated with reduced DSS only |
| **Ghadjar et al. (2015)** | **Pre-RT** | WL ≥ 10 % within 6 months | OS, LRRFS, DMFS, CSS | ↓ (all) | p ≤ 0.002 | Consistent negative prognostic impact |
| **Han et al. (2021)** | **During-RT** | WL ≥ 5.8 % during RT | OS, CSS | ↑ (both) | p < 0.05 | Greater WL associated with better survival (interpret with caution) |
| **Moon et al. (2016)** | **Pre-RT (BMI)** | BMI < 18.5 kg/m² before RT | OS, CSS, PFS | ↓ (all) | p < 0.001 | Low pre-BMI indicates worse survival |
| **Moon et al. (2016)** | **During-RT** | ΔBMI (pre vs 2 mo post-RT) | OS, CSS, PFS | ↔ | ns | No impact of BMI change during RT |
| **Yu-Hsuan Lin et al. (2015)** | **During-RT** | WL ≥ 5 % during RT | OS, DSS | ↔ | ns | No association with survival |
| **Xiang Lin et al. (2022)** | **During-RT** | ΔBMI > 1 kg/m² during VMAT | OS, DFS, D-FFR | ↓ (all) | p < 0.001 | Independent predictor of poor outcomes |
| **Cho et al. (2012)** | **Post-RT** | WL ≥ 10 % within 6 months after RT | DFS | ↓ | p = 0.036 | Post-RT WL associated with poorer DFS |
| **Ottosson et al. (2014)** | **During-RT** | WL ≥ 10 % (start RT → 5 months) | OS | ↔ | ns | No significant effect on OS |

**Legend:** Arrows indicate direction of association:
↓ = worse survival with higher WL; ↑ = better survival with higher WL; ↔ = no association.
Abbreviations: OS = overall survival; DSS = disease-specific survival; DFS = disease-free survival; CSS = cancer-specific survival; LRRFS = locoregional recurrence-free survival; DMFS = distant metastasis-free survival; D-FFR = distant failure-free rate; VMAT = volumetric modulated arc therapy; ns = not significant.

***Supplementary*** ***Table S3:*** *Overview matrix summarizing the prognostic associations between weight loss (WL) timing and survival outcomes across the included studies.*

| **Author, year** | **Pre-WL** | **WL during RT** | **Post-WL** | **ΔBMI** | **OS** | **DSS / CSS** | **DFS** | **Other (DMFS, LRRFS, D-FFR)** |
| --- | --- | --- | --- | --- | --- | --- | --- | --- |
| **Langius et al. (2013)** | ↓ (>10 %) ** | ↓ (DSS) | – | – | ↓ | ↓ | – | – |
| **Ghadjar et al. (2015)** | ↓ (≥10 %) ** | ↔ | – | – | ↓ | ↓ | ↓ | ↓ (DMFS, LRRFS) |
| **Han et al. (2021)** | – | ↑ (apparent) | – | – | ↑ | ↑ | – | – |
| **Moon et al. (2016)** | ↓ (BMI < 18.5) | ↔ | – | ↔ | ↓ | ↓ | ↔ | – |
| **Yu-Hsuan Lin et al. (2015)** | ↔ | ↔ | – | – | ↔ | ↔ | – | – |
| **Xiang Lin et al. (2022)** | ↔ | – | – | ↓ (> 1 kg/m²) ** | ↓ | – | ↓ | ↓ (D-FFR) |
| **Y. Cho et al. (2012)** | – | – | ↓ (≥10 %) ** | – | – | – | ↓ | – |
| **Ottosson et al. (2014)** | ↓ (BMI > 25 better) | ↔ | – | – | ↑ | – | – | – |

**Legend:** Arrows indicate the direction of association: ↓ = worse survival with higher WL or lower BMI; ↑ = better survival; ↔ = no association; – = not assessed. ** = statistically significant.
Abbreviations: OS = overall survival; DSS = disease-specific survival; CSS = cancer-specific survival; DFS = disease-free survival; DMFS = distant metastasis-free survival; LRRFS = locoregional recurrence-free survival; D-FFR = distant failure-free rate.
