# Supplementary material for: Prognostic impact of weight loss during radiation therapy in patients with head and neck cancer: A systematic review
Source: Nutr Health. 2026 Feb 13;32(4):1169–78. doi: 10.1177/02601060261419743 (PMC13338475; doi:10.1177/02601060261419743)
Supplement: sj-docx-2-nah-10.1177_02601060261419743 - Supplemental material for Prognostic impact of weight loss during radiation therapy in patients with head and neck cancer: A systematic review [file sj-docx-2-nah-10.1177_02601060261419743.docx]

**Appendix A – Electronic Search Strategy**

**Database searched:** PubMed/MEDLINE (via NCBI) and Cochrane Library
**Date of last search:** November 30, 2024
**Limits:** Human studies, English language, publication year ≥ 2012
**Search fields:** Title/Abstract and MeSH Terms

**Search string:**

(("head and neck neoplasms"[MeSH Terms]) OR ("head and neck cancer"[Title/Abstract]) OR ("HNSCC"[Title/Abstract]))

AND

(("radiotherapy"[MeSH Terms]) OR ("chemoradiotherapy"[Title/Abstract]) OR ("radiation therapy"[Title/Abstract]))

AND

(("weight loss"[MeSH Terms]) OR ("body mass index"[MeSH Terms]) OR ("BMI change"[Title/Abstract]) OR ("malnutrition"[Title/Abstract]) OR ("nutritional status"[Title/Abstract]))

AND

(("survival"[MeSH Terms]) OR ("prognosis"[MeSH Terms]) OR ("overall survival"[Title/Abstract]) OR ("disease-free survival"[Title/Abstract]))

**Additional steps:**

- Manual screening of bibliographies from key papers and reviews.
- Cross-checking in Cochrane Library using equivalent MeSH terms.
- No additional filters (sex, age, tumor site) were applied to maximize sensitivity.
